# Epigenetic Changes in the HTR8 and 3A-sub E placental Cell Lines Exposed to Bisphenol A and Benzyl Butyl Phthalate

**DOI:** 10.3390/toxics12090659

**Published:** 2024-09-09

**Authors:** Christian Litton, Paula Benny, Luca Lambertini, Yula Ma, Jonathan Riel, Rodrigo Weingrill, Johann Urschitz, Jia Chen, Men-Jean Lee

**Affiliations:** 1 Department of Obstetrics and Gynecology, Maine Medical Center, Portland, ME 04102, USA; 2 Department of Obstetrics and Gynecology, John A. Burns School of Medicine, University of Hawaii, Honolulu, HI 96822, USA; 3 The Diabetes, Obesity and Metabolism Institute, Icahn School of Medicine at Mount Sinai, New York, NY 10029, USA; 4 Department of Environmental Medicine and Public Health, Icahn School of Medicine at Mount Sinai, New York, NY 10029, USA

**Keywords:** bisphenol A, benzyl butyl phthalate, epigenetics, placenta, plasticizer

## Abstract

Objective: Bisphenol A and phthalate are known endocrine disruptors and capable of inducing epigenetic changes in the human population. However, their impact on the placenta is less well studied. Our objective was to measure the effect of exposure to bisphenol A and benzyl butyl phthalate in first-trimester HTR8-SVneo and third-trimester 3A-sub E trophoblast cells by profiling the DNA methylation pattern of the imprinting control region of the IGF2 (insulin-like growth factor) and H19 genes. Methods: Human placental HTR8-SVneo and 3A-sub E cell lines were treated with two sub-lethal concentrations of bisphenol A and benzyl butyl phthalate. Demethylating agent, 5-azacytidine, was used as a positive control. Cells were harvested on post-treatment days 1 and 4. The methylation profile of six CpG dinucleotide sites, part of the CTCF 6 binding site of the IGF2/H19 imprinting control region, was determined by pyrosequencing. Results: In the first-trimester HTR8-SVneo cell line, we observed a significant increased methylation of the CpG sites 3, 4 when treated with a high concentration of bisphenol A or benzyl butyl phthalate while increased methylation at site 6 for both high and low dose treatment on day 4. Demethylation of the CpG sites 1, 4, and 6 was observed when treated with 5-azacytidine on day 4. In the third-trimester 3A-sub E cell line, no significant changes in the methylation profile were observed under any treatment conditions. Conclusions: The results of this study demonstrate the capability of epigenetic changes in human placenta cells induced by bisphenol A and benzyl butyl phthalate. The observed methylation changes only in the first-trimester HTR8-SVneo cells phthalate may reflect a window of epigenetic susceptibility related to these environmental toxicants.

## 1. Introduction

Bisphenol A (BPA) and benzyl butyl phthalate (BBP) are industrial plasticizers found in a wide variety of items ranging from dental fillings, canned foods, and single-use water bottles to textile flame retardants. BPA and BBP are two of the most widely recognized endocrine disrupters that have also been linked to immune dysfunction, neurobehavioral disorders, and common reproductive disorders such as uterine fibroids, prostate hypertrophy, polycystic ovarian syndrome, and even breast cancers [1,2,3,4,5,6]. Plastic products and other popular plasticizers are ubiquitously found in the modern world and have been reported to break down and become absorbed into human tissues. Microplastics have been found in the human gut and pregnancy tissues such as placentas and breastmilk, but the developmental effects of plasticizers on the maturation of human placentas have yet to be elucidated. In addition, fetal exposure and the accumulation of plasticizers in the fetal and placental compartments have also been demonstrated but their role in inducing epigenetic toxicity continues to be speculative [7]. Plasticizers tend to be labelled as endocrine-disrupting chemicals (EDCs) due to their estrogenic properties, especially BPA, which is widely known to bind to estrogen receptors (ER-α and ER-β). Although the full extent of cellular level impact of microplastic particle accumulation in the human placenta has not been fully elucidated, both plasticizer compounds of interest are purported to exert their influence through epigenetic mechanisms such as methylation or histone modifications [8,9]. The human placenta is a vital organ which exhibits developmental changes to support the physiological growth of an embryo to the mature fetus. As the placenta changes from an invasive tissue type into a vascular organ, the proportions and functions of the various cell types which make up a placental cotyledon evolve from the first trimester to the third [10,11]. The functional unit of the placental villus is characterized by the maturation of trophoblast cells which regulate fetal growth over the course of pregnancy through a variety of genes including IGF2. The burgeoning field of environmental epigenetics has identified the IGF2 locus as being one of those epigenetically labile gene clusters with metastable alleles. Therefore, we aimed to determine if BPA and BBP can induce epigenetic changes in first-trimester compared to third-trimester trophoblast cell lines. The IGF2/H19 gene cluster is particularly interesting as IGF2/IGF2R/H19 were the first imprinted genes discovered which critically affect fetal size and metabolism [12]. We hypothesized that the imprinting control regions of growth-regulatory genes in the well-characterized IGF2/H19 gene cluster might be differentially susceptible to BPA or BBP in first-trimester compared to third-trimester cultured placental trophoblast cells. 

**Figure 1:**
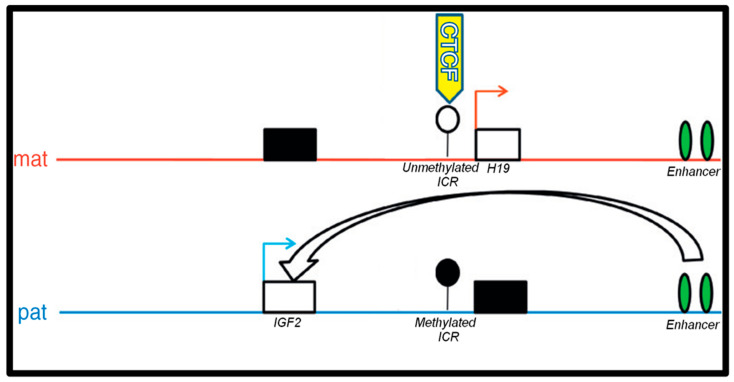
The IGF2/H19 imprinting control region. The CTCF protein binding to the unmethylated allele promotes H19 expression from the maternal allele. CTCF 6 methylation allows for the interaction of the enhancer downstream to H19 with the IGF2 promoter leading to IGF2 expression from the paternal allele [18,19].

**Figure 2:**
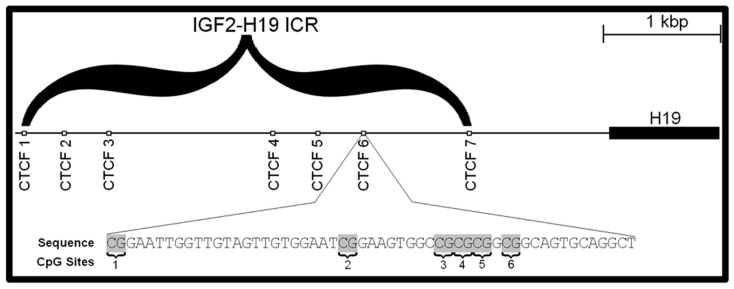
The CTCF 6 site of IGF2/H19 imprinting control region. The IGF2/H19 ICR is composed of seven CTCF sites of which only CTCF 6 has been shown to display an allele-specific profile. The other CTCF sites are considered to be derived from duplications of the CTCF 6 site. CpG site 6 is located further upstream [16].

An imprinting control region (ICR) is a chromosomal region that acts, in a methylation-sensitive way, to determine whether imprinted genes are expressed or not according to the parent from which the gene derived. The IGF2/H19 ICR, located upstream of the H19 gene, controls the transcription of both genes in a parental-specific manner [13,14]. Alterations in the methylation of the IGF2/H19 ICR have been associated with genetic syndromes including Russell–Silver [15]. The expression of the IGF2 and H19 genes appears to be controlled through the binding of the transcriptional factor CTCF (Figure 1). CTCF is an 11-zinc finger protein that acts as a transcriptional repressor primarily by altering the three-dimensional structure of the chromatin. The IGF2/H19 ICR is composed of seven CTCF binding sites, which control the transcription of the IGF2 and H19 genes (Figure 2). The CTCF 6 binding site, which contains six CpG dinucleotides, has been shown to display an allele-specific pattern and was chosen as the area of interest for this study [16]. The methylation pattern of the six CpG dinucleotides of the CTCF 6 binding site affects the ability of the CTCF protein to bind and, therefore, regulate downstream transcription [17,18]. Therefore, we also posited that BPA and BBP exposure in the two placental cell lines might lead to different methylation patterns of the CpG sites within the CTFC 6 binding site on the IGF2/H19 ICR, similar to observations from other human cell lines, potentially explaining the underlying mechanisms of some of the observed disruptions in fetal development after BPA/BBP exposure.

## 2. Materials and Methods

### 2.1. Cell Lines and Exposure

After the Institutional Review Board designated this research as non-human subjects research, the HTR8-SVneo transformed placental cell line (first trimester) and 3A-sub E trophoblast cells (third trimester) were obtained as a gift from Dr. Seth Guller (Yale School of Medicine, Department of Obstetrics, Gynecology and Reproductive Sciences). These trophoblast cells were cultured in RPMI 1640 (Invitrogen, Inc. Gaithersburg, GA, USA) medium. Medium was supplemented with 10% charcoal-stripped fetal bovine serum (Invitrogen, Inc., Gaithersburg, GA, USA) and penicillin-streptomycin. The first-trimester HTR8-SVneo and third-trimester 3A-sub E trophoblast cells were then treated over a four-day period with BPA, BPP, 5-azacytidine (AZA), or control media. AZA is a chemotherapeutic agent that is known to cause demethylation. Fully methylated, commercially available human Jurkat cells were used as a positive treatment control [19]. Additionally, unmethylated experimental controls were obtained from amplifying an amplicon encompassing the CTCF 6 site by standard PCR.

A four-day incubation was chosen to mimic long-term, chronic exposure while avoiding sub-culturing of cells. Two dosages of the compounds were chosen to evaluate for the presence of a dose-dependent response. The dosages for BPA and BBP were chosen based on data from titration experiments demonstrating non-lethal concentrations with minimal cellular disruption. Additionally, the concentrations were within the range of previously published doses used to study BPA and BBP exposure in placental cell lines [2,20]. HTR8-SVneo and 3A-sub E cells were cultured in six-well plates that were seeded with 10^5 cells for 48 h with RPMI 1640 media. Cells were treated with 0, 1 nM, and 100 nM BPA; 0, 10 nM, and 1000 nM BBP; and 0, 5 nM, and 500 nM AZA. Jurkat cells were treated with 0, 5 nM, and 500 nM AZA, respectively. Fresh media containing the respective agents were changed on experimental day 0 and day 2. Cells were harvested on experimental day 1 and day 4. All experimental and control conditions were performed in triplicate.

### 2.2. DNA Isolation

Cells were harvested by trypsinization and centrifugation. Media was discarded and cell pellets were washed with phosphate-buffered saline. Total DNA was extracted and purified using the QIAamp DNA Mini Kit (Qiagen Inc., Valencia, CA, USA) according to the manufacturer’s protocol. DNA quantity and purity was assessed using the ND-2000 spectrophotometer (Thermo Fisher Scientific, Wilmington, DE, USA), aliquoted, and stored at −80 °C until all experimental samples could be run simultaneously.

### 2.3. Bisulfite Conversion

Complete bisulfite conversion of unmethylated cytosine residues to uracil and cleanup of the DNA was then performed for methylation analysis using the EpiTect Bisulfite Kit (Qiagen Inc., Valencia, CA, USA) according to the manufacturer’s protocol.

### 2.4. Pyrosequencing

DNA sequencing of the six CpG dinucleotides, which are part of the CTCF 6 binding site, was performed using the PyroMark Q24 (Qiagen Inc., Valencia, CA, USA) instrument according to the manufacturer’s instructions. DNA sequencing was used to determine the methylation pattern of the six CpG dinucleotides of the CTCF 6 binding site. (Sequences analyzed: GGTAYGGAATTGGTTGTAGTTGTGGAATYGGAAGTGGTYGYGYGGYGGTAGTGTAGGTT).

### 2.5. Statistical Analysis

The differences in methylation of the six CpG sites for each test condition by cell type on day 1 and day 4 of treatment were compared using ANOVA, with *p* < 0.05 considered significant. Post hoc analysis with the Tukey–Kramer HSD test was used to determine which differences were significant when compared to each respective control cell types at each time point, at each of the six CpG sites, with alpha = 0.05.

## 3. Results

**Figure 3:**
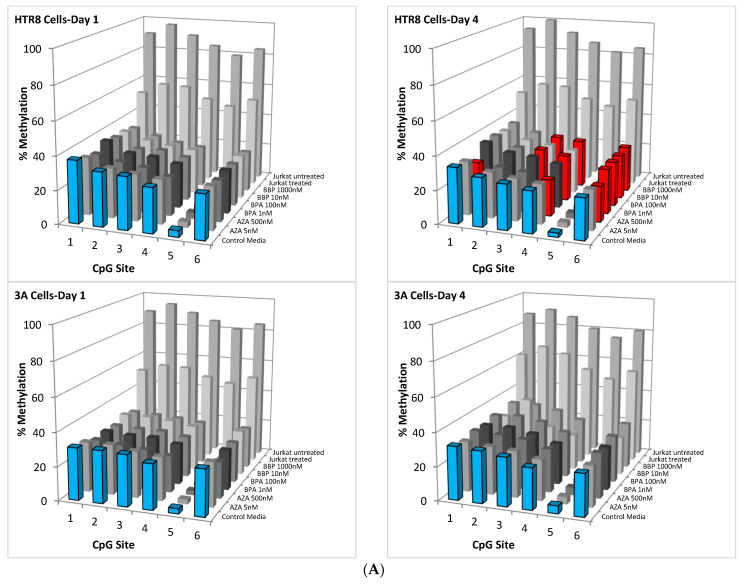

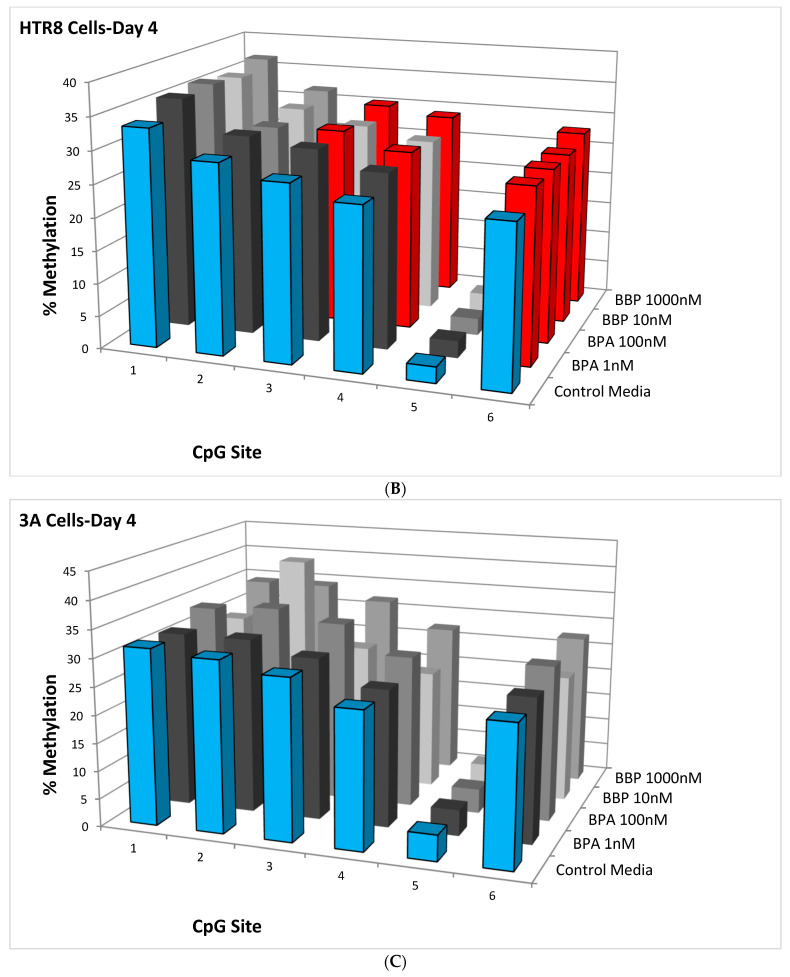
Methylation analysis of the six CpG dinucleotides across CTCF 6 of the IGF2/H19 Imprinting Control Region. (**A**) Overview of the methylation of the CpG sites 1–6 from the HTR8 and 3A-sub E cell lines at days 1 and 4 following treatment with control media, AZA, BPA, and BBP. The fully methylated control is represented by the untreated Jurkat cells. Blue bars represent the control media. Red bars indicate CpG sites with significant differences from the control media. (**B**) Detail of the differential methylation of the CpG sites 1–6 from the HTR8 cell line at day 4 following treatment with control media, BPA, and BBP. The differences in methylation following treatment were significant (red bars) when compared to control treatment (blue bars) for each indicated CpG site (ANOVA followed by Tukey–Kramer HSD analysis, *p* < 0.05). (**C**) Detail of the differential methylation of the CpG sites 1–6 from the 3A-sub E cell line at day 4 following treatment with control media, BPA, and BBP. The differences in methylation following treatment were not significant when compared to control treatment (blue bars) for any of the CpG sites (ANOVA followed by Tukey–Kramer HSD analysis, *p* = NS).

First-trimester HTR8-SVneo cells demonstrated significant demethylation by AZA by day 4 of treatment at the CpG sites 1, 4, and 6 as a positive control. Interestingly, on day 4 of treatment, HTR8-SVneo cells demonstrated a significant increase in methylation of the CpG sites 3, 4, and 6 following exposure to BBP at 1000 nM and BPA at 100 nM (Figure 3A,B). Furthermore, BPA treatment at 1 nM and 100 nM, and BBP treatment at 10 nM and 1000 nM all increased methylation at the CpG site 6 on day 4 (Figure 3A,B; ANOVA followed by Tukey–Kramer HSD analysis, *p* < 0.05). In contrast, third-trimester 3A-sub E cells did not demonstrate any significant changes in methylation after treatment with AZA, BPA, or BBP at any of the corresponding CpG sites at any time point (Figure 3A,C; ANOVA followed by Tukey–Kramer HSD analysis, *p* = NS).

First-trimester HTR8-SVneo placental trophoblast cells demonstrated methylation changes in the IGF2/H19 ICR in response to exposure to the plasticizers BPA and BBP. A number of CpG dinucleotides on the CTCF 6 binding site of IGF2/H19 ICR were found to have increased levels of methylation in a dose-dependent fashion following BPA or BBP treatment. This observation is in contrast to a decreased methylation pattern with exposure to BPA or BBP that had been previously reported in prostate and breast cancer cell lines [21]. Additionally, since the effects of the BPA and BBP were not seen until day 4 of the treatment protocol, this suggests the potential effect of chronic exposure.

## 4. Discussion

The study of epigenetics investigates the heritable changes that are not the result of alterations to the DNA sequence. Often, alterations in gene expression are the result of an epigenetic occurrence rather than a new mutation. Genomic imprinting is a type of epigenetic phenomenon that refers to the expression of genes in a parent-of-origin manner. Although most human genes are expressed in a biallelic fashion (i.e., both maternal and paternal alleles are concomitantly expressed), imprinted genes are expressed in a monoallelic manner [13]. Imprinted genes have been shown to be closely involved in the normal development of the fetoplacental unit. Alterations of genomic imprinting are well described and occur in multiple genetic syndromes, such as Prader–Willi, Angelman, Beckwith–Wiedemann, and Russell–Silver [15,22,23]. DNA methylation is an epigenetic mechanism whereby the expression of a gene is altered by disrupting DNA transcription and involves a biochemical process in which a methyl group is added to a cytosine residue. BPA and BBP have been shown to alter DNA methylation patterns in certain cell signaling genes [24,25]. For example, in a model of prostate cancer, neonatal rats exposed to BPA demonstrated hypomethylation of CpG islands [26]. Additionally, treatment of MCF7 breast cancer cells with BBP has been shown to result in the demethylation of an estrogen receptor, alpha promoter-associated CpG islands [27]. Furthermore, BPA has been shown to alter methylation patterns in the ICR of embryonic mouse models [28]. The human IGF2 and H19 genes are involved in the regulation of growth and mitogenic activity and are expressed in a monoallelic manner. Typically, IGF2 expression originates from the paternal allele while H19 is expressed from the maternal allele. However, disruptions in the expression of these genes have been linked to various adverse perinatal outcomes, including fetal growth restriction and abnormal neurodevelopmental outcomes [22,29]. Therefore, the perturbation of the IGF2/H19 ICR in our first- and third-trimester placental trophoblast models suggest that exposure to BPA and BBP has the potential to alter fetal development and growth, as well as fetal programming of adult disease.

The combination of our observations that the methylation pattern of the CTCF 6 binding site of the IGF2/H19 ICR in the 3A-sub E cell line is insensitive to treatment with the plasticizers BPA and BBP and the observation that 3A-sub E cells were insensitive to the potent demethylating agent AZA suggests that there may be a developmental window of susceptibility for first-trimester trophoblast cells (e.g., HTR8-SVneo cells) to be affected by plasticizers that is absent by the third trimester of pregnancy.

Interestingly, Jurkat cells remained approximately 50% methylated following AZA treatment. AZA was unable to completely demethylate the CTCF 6 binding site in these cells although AZA has been described to induce promoter demethylation [30]. This suggests the existence of a specific system that protects this region from complete loss of methylation or that higher doses of AZA may be needed for complete demethylation.

Our findings are similar to other placental studies looking into phthalate exposure on epigenetic outcomes [31]. Further research from our group has also demonstrated a low level of IGF2 and H19 gene expression in these placenta cell lines (manuscript in preparation). Therefore, the accompanying methylation profiles noted in this study may reflect the relative transcriptional inactivation of IGF2 and H19 genes during placental development.

The use of two distinct placental cell lines from first and third trimesters to assess the effects of plasticizers in the developing placenta represents a strength of this study. In addition, the experimental treatments at low and high concentrations of BBP and BPP showed the dose-dependent effects of these chemicals on placental cells. Finally, the assessment of methylation changes across two time points provided a model for short- and longer-term exposure on placental cells, as shown in Supplementary Figure S1.

Therefore, the regulation of expression of the IGF2 and H19 genes in the placenta might be more complicated than previously thought. The methylation pattern of these genes, as well as the resistance of these genes to demethylation, points to a multifaceted interplay of dose response, duration of exposure, developmental changes, and cell-type specificity. Future studies may build on the results of this study to elucidate the role that the IGF2 and H19 genes play in the pathophysiology of adverse pregnancy outcomes, for example, intrauterine growth restriction [32]. In addition, further research should also include modeling the effects of microplastic particles on first- and third-trimester trophoblast cell lines to determine whether microplastics versus plasticizers have stronger effects on fetal growth, and ultimately maternal and neonatal health.

The increase in accumulation of microplastics in human placentas over the past decades highlights the importance of expanding our understanding of the effects of plasticizer chemicals on the developing placenta and fetus [33]. Given the potential lifelong consequences of epigenetic alterations in the fetal profile, a clear understanding of how these compounds instigate changes at the level of gene regulation may assist in directing lifestyle modifications to optimize pregnancy outcomes.

## 5. Conclusions

This study provides new knowledge of epigenome–environment interactions in fetal development. In particular, we have demonstrated a susceptibility of the human placenta to epigenetic modifications in early pregnancy following exposure to common plasticizers compared to late term placentas. Further research is needed to provide a better understanding of the role that environmental exposures to EDCs play during the different stages of pregnancy, in the hope of enhancing beneficial outcomes for fetal growth and improving child health outcomes.

## Data Availability

The data that support the findings of this study are available from the corresponding author, upon request.

## Supplementary Materials

The following supporting information can be downloaded at: https://www.mdpi.com/article/10.3390/toxics12090659/s1, Figure S1: Summary.
