# Supplementary material for: Epigenetic Changes in the HTR8 and 3A-sub E placental Cell Lines Exposed to Bisphenol A and Benzyl Butyl Phthalate
Source: Toxics. 2024 Sep 9;12(9):659. doi: 10.3390/toxics12090659 (PMC11435974; doi:10.3390/toxics12090659)
Supplement: Supplementary file 1 [file toxics-12-00659-s001.zip › toxics-3159152-supplementary.pdf]

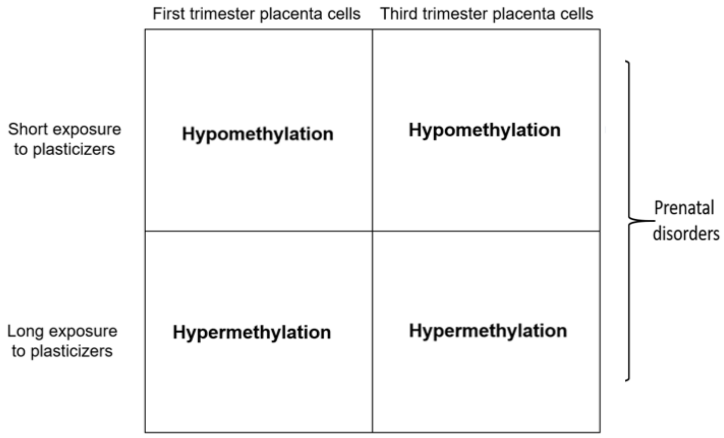

Supplementary Figure S1. Summary.
